# Complete genome sequence of *Pseudoxanthomonas koreensis* strain TDY-1, an odor-reducing bacterium isolated from livestock wastewater

**DOI:** 10.1128/mra.01027-24

**Published:** 2024-12-31

**Authors:** Kook-Il Han, Young Ho Nam, Eui-Jin Kim

**Affiliations:** 1 Using Technology Development Department, Nakdonggang National Institute of Biological Resources (NNIBR), Sangju, South Korea; The University of Arizona, Tucson, Arizona, USA

**Keywords:** *Pseudoxanthomonas koreensis* TDY-1

## Abstract

We report the complete genome sequence of *Pseudoxanthomonas koreensis* TDY-1*,* an odor-reducing strain isolated from livestock wastewater on a pig farm. Its genome consists of a 2.97-Mb chromosome.

## ANNOUNCEMENT

Odors are released from numerous industrial sources, including livestock farms, wastewater treatment facilities, paint shops, and various chemical industries (1). Livestock farm odors are characterized by a range of compounds, including ammonia, hydrogen sulfide, volatile fatty acids, and *p*-cresol (2).

*Pseudoxanthomonas koreensis* TDY-1 was isolated from livestock wastewater on a pig farm (36°25′5″N, 128°18′59″E), Republic of Korea, in January 2023 by the dilution plating method. The livestock wastewater was collected using 20-L sampling bottles. The sample was serially diluted in saline (0.85% sodium chloride) and cultured on R2A agar (Difco, USA) for 5 days at 30°C under aerobic conditions. Single colonies were isolated on R2A agar plates. TDY-1 was cultured in tryptic soy broth (Difco, USA) at 30°C with agitation at 120 rpm for 2 days. The culture was concentrated to an optical density of 4 (OD_600_) and used as the inoculum. Sterilized 1 L bottles were prepared by adding 99 mL of swine manure. The experimental group was treated with 1% (vol/vol) TDY-1 culture, while the control group received 1% (vol/vol) sterile water. A tube was inserted into the bottle and sealed using a clamp, followed by incubation at 30°C for 1 day. Ammonia concentration in the headspace was quantified using a gas detection tube (Gastec, Japan) with a sampling volume of 100 mL. Experiments were conducted in triplicate, and the ammonia reduction efficiency of the experimental group was 10% ± 2% lower compared to the control group.

Strain TDY-1 was cultivated on R2A agar (Difco, USA) at 30°C for 5 days. Multiple colonies were transferred and suspended in Maxwell RSC Tissue DNA kit buffer (Promega, USA) for genomic DNA isolation. For long-read sequencing, DNA was fragmented utilizing a g-TUBE device (Covaris, USA) and prepared with the 15-kb SMRTbell template kit (Pacific Biosciences, USA) and sequenced on a Sequel II system (size cutoff: 7–12 kb). Supplementary Illumina short-read sequencing was conducted with a TruSeq Nano DNA Kit (Illumina, USA) on the NovaSeq platform, generating 151-bp paired-end reads. Library preparation and sequencing were performed by Macrogen (South Korea). The 94,217 total reads (read *N*_50_, 8,419 bp) produced using the PacBio SequelII platform were assembled; sequencing on the Illumina platform produced 18,862,383 total reads. *De novo* assembly of the long reads (PacBio Sequel II) was carried out using the HGAP4 software (3). Low-quality reads and adapters were removed using Trimmomatic version 0.38 (4), and Pilon version 1.21 (5) was used for error correction. Assembly quality was evaluated through BUSCO version 5.1.3 (6) analysis. Gene prediction was performed using Prokka version 1.14.6 (7). The adapter overhang sequence was removed to produce sub-reads using SMRT Link (version 13.0.0.207600). Circular maps of each contig were visualized using Circos version 0.69.6 (8), resulting in the identification of a single circular chromosome measuring 2.97 Mbp (Table 1). Default parameters were used for all software unless otherwise specified.

**TABLE 1:** Summary of the assembly and annotation statistics for *Pseudoxanthomonas koreensis* TDY-1

Parameter data	
Genetic element	Chromosome
Length (bp)	2,978,518
GC content (%)	69.5
No. of coding sequences	2,618
No. of rRNAs	3
No. of tRNAs	45
Sequencing depth (×)	232
GenBank accession no.	CP169056

CDS prediction and annotation were performed using the NCBI Prokaryotic Genome Annotation Pipeline version 6.8 with GeneMarkS-2+ (9). We identified potential odor-reducing genes within the genome, including a gene cluster involved in the downregulation of nitrogen (*nirD* and *petC*), sulfate (*sta*, *sir*, and *CysH*), and fatty acid metabolism (*fadD*, *fadA*, *fadB*, *fadE*, and *fadJ*), in addition to a phenol hydrolase gene (*pheA*).

## Data Availability

The genome sequence and raw sequencing reads for strain TDY-1 were deposited under GenBank accession number CP169056, BioProject accession number PRJNA1151831, and BioSample accession number SAMN43324457. The raw reads have been deposited in the Sequence Read Archive (SRA) under SRA accession numbers SRR30790981 and SRR30790980. *Pseudoxanthomonas koreensis* strain TDY-1 has been deposited in the Korean Collection for Type Cultures under accession number KCTC 15890BP.
